# From Proteome to miRNome: A Review of Multi-Omics Ocular Allergy Research Using Human Tears

**DOI:** 10.3390/ijms27020671

**Published:** 2026-01-09

**Authors:** Esrin Aydin, Serap Azizoglu, Luke Chong, Moneisha Gokhale, Cenk Suphioglu

**Affiliations:** 1 Neuro Allergy Research Laboratory (NARL), School of Life and Environmental Sciences, Deakin University, Geelong, VIC 3216, Australia; 2 School of Medicine (Optometry), Deakin University, Geelong, VIC 3216, Australia; 3 Institute for Mental and Physical Health and Clinical Translation (IMPACT), Deakin University, Geelong, VIC 3216, Australia; 4 Centre for Sustainable Bioproducts (CSB), Deakin University, Geelong, VIC 3216, Australia

**Keywords:** allergic rhinoconjunctivitis, angiogenesis, inflammation, ocular allergy, wound healing

## Abstract

Ocular allergy (OA) is a subtype of seasonal allergy that causes symptoms of itchiness, redness, swelling and irritation of the ocular surface and eyelids, often triggering allergy-induced eye rubbing and sustained inflammation for up to six months of the year during peak allergy season. These symptoms, coupled with reduced sleep quality, impaired daily productivity and decreased mood, highlight a significant yet underrepresented disease burden. Recent advances in tear-based multi-omics have enabled detailed characterisation of OA-associated biochemical changes on the ocular surface, highlighting human tears as a promising biospecimen for diagnostic biomarker and therapeutic target research. This review discusses emerging proteomic, lipidomic, metabolomic and miRNA findings comparing OA sufferers with healthy controls, and, where relevant, with comorbid conditions such as dry eye disease and keratoconus. Differential expression patterns across these analytes implicate key pathways involved in immune response, wound healing, angiogenesis, inflammation, oxidative stress and return to homeostasis on the ocular surface. By integrating these data into a stepwise model of OA biopathway activation, this review outlines candidate biomarkers and highlights methodological advances that may support translation of tear multi-omics into clinical tools for OA management.

## 1. Introduction

Ocular allergy (OA) is an underrepresented allergic condition in rapidly developing multi-omics literature, despite significant Quality of Life effects such as reduced sleep quality, impaired daily productivity and poor mood [1]. Approximately one in four Australians suffer from seasonal allergies, the defining symptoms of which include itchiness, irritation, redness and swelling of the eyelids and conjunctiva [2]. This article aims to collate the emerging literature on human tear multi-omics research within OA research.

Grading methods used to assess signs and symptoms of OA in a clinical setting for diagnosis are subjective in nature and therefore may be liable to examiner bias. While still useful for determining OA diagnosis, subjective grading may not be accurate for determining the severity of OA and therefore may incorrectly influence the strength of treatments prescribed [3]. An alternative for subjective measures such as grading and patient-reported symptoms would be using objective biochemical markers such as proteins, lipids, metabolites and miRNAs found in the tears to classify different levels of severity of OA. These biomarkers may offer a more objectively quantifiable and reproducible method of assessing ocular surface health, reducing the impacts of bias. Thus, providing a deeper insight into the molecular changes occurring on the ocular surface in ocular disorders such as OA and its common comorbidities.

## 2. Comorbidities

Biochemical changes in the tear film have been well documented in dry eye disease (DED) [4]. DED is defined by the Tear Film and Ocular Surface Society Dry Eye Workshop as “a multifactorial, symptomatic disease characterised by a loss of homeostasis of the tear film and/or ocular surface, in which tear film instability and hyperosmolarity, ocular surface inflammation and damage, and neurosensory abnormalities are etiological factors” [5]. DED was shown to be a common comorbidity of OA in a meta-analysis from 2022 that combined findings from six studies across Asia, Europe and Africa, spanning a total of 1932 OA sufferers [6]. Among them, 47.2% of OA sufferers also suffered from DED [6]. It is theorised that this comorbidity is likely caused by overlapping inflammatory pathology and a potential additional link between tear film biochemistry [6]. Namely, thinning of the tear film lipid layer and compositional changes may contribute to the comorbidity of DED and OA by increasing tear evaporation rates and therefore increasing inflammation and irritation on the ocular surface. A study assessing lipidomic changes to the tear film due to OA and DED in conjunction has never been undertaken before, but would be insightful for developing a deeper understanding of the potential biochemical link between the two comorbidities beyond the existing literature.

Another comorbidity of OA includes keratoconus, a condition linked to OA through physical changes to the cornea as a result of chronic eye rubbing. Corneal changes due to chronic eye rubbing have been shown to occur in keratoconus patients, causing permanent changes to collagen matrices of the cornea that could, in turn, cause decreased visual acuity [7]. Keratoconus is a corneal ectatic disorder in which the cornea begins to droop and lose structure over time, causing progressive visual loss and damage [8]. A study of keratoconus patients by Weed et al. showed that 48% of patients rubbed their eyes frequently, leading to poorer prognosis and visual health, as well as showing that 30% of keratoconus patients experienced seasonal allergies as a comorbidity [9]. This increased frequency of eye rubbing has been shown to be problematic for the ocular surface, negatively affecting the biochemistry of the tear film and permanently altering the physical structure of the cornea [10]. As it stands, there is insufficient research into the longitudinal effects of allergy-induced eye rubbing and inflammation on the ocular surface and on altered tear biochemistry. By identifying biomarkers of OA for therapeutic and diagnostic development, the incidence of allergy-induced eye rubbing may be decreased through more effective itch-relieving treatment options, therefore preventing potentially irreversible physical and biochemical changes to the ocular surface, as shown in keratoconus and DED.

## 3. Developments in Multi-Omics Analysis

Currently, the literature does not paint a cohesive image of the biochemical changes occurring on the ocular surface of OA sufferers. Omics research has increased in popularity in the last 10 years with the advent of technologies such as high-throughput sequencing, point-of-care diagnostic tools, mass-spectrometry-based technologies and advanced bioinformatics techniques for analysis of large, complex datasets. These tools have enabled characterisation of the unique molecular composition of tears, identifying significant changes in proteins, lipids, metabolites and nucleic acids that accompany allergic inflammation. As a result, tear-based multi-omics is rapidly expanding our understanding of ocular surface immunology in the context of OA.

Tear analysis in particular has seen considerable development, with extraction and analytical methods evolving to improve analyte recovery, reduce sample volume requirements and enhance analyte detection limits. Simplified tear analysis methods such as IgE testing to distinguish OA patients from healthy controls have reflected a significant increase in sensitivity of testing methods over time [11,12,13]. IgE analysis methods from 26 years ago utilised complex analytical methodology comprising combined immunoblotting and Enzyme-Linked Immunosorbent Assay (ELISA) approaches [13]. Emerging research by Thomas et al. and Shang et al. emphasise the progress in this field through testing point-of-care OA diagnostic devices that use 10 µL of tear fluid collected via glass microcapillary tubes [11,12]. Findings from these studies indicate remarkable technological advancement, with respective Area Under the Curve (AUC) values of 0.893 and 0.896 for prediction of allergy via IgE analysis [11,12]. While seemingly robust, diagnostics are not the prime focus of multi-omics analysis of human tears, with other applications existing, including therapeutic development, biomarker discovery and investigations of the long-term allergy impact on ocular surface health [14,15].

Prior method development in tear fluid-omics research has largely centred around samples collected via Schirmer strips due to high protein recovery [16,17]; however, protein recovery and subsequent analytical methods may vary greatly with unknown impacts on resultant data [17]. A discovery study utilising Olink analysis of 92 target analytes from a Schirmer strip highlighted the variability of protein detection dependent on the distance of the punch from the head of the strip (which is in direct contact with the conjunctival epithelium) versus the end of the strip [17]. The end of the strip was hypothesised to have reduced detectable protein quantities due to the influence of the molecular weight of the protein on its ability to move up the strip [17]. This effect is not seen in glass microcapillary flow, as the specimen is uniformly expelled via centrifugation prior to analysis. This may suggest that tear samples collected via glass microcapillary are more representative of the tear film due to minimal interference from the collection method, reduced reflex tearing and decreased cellular contamination [18]. Furthermore, recent advancements in tear fluid analysis using samples collected via glass microcapillary suggest that 500–800 proteins are able to be detected via Liquid Chromatography–Mass Spectrometry (LC-MS/MS) in as little as 0.5 μL of tear fluid in 2 h [18] as opposed to traditional methods, which may take between 5 and 8 h to analyse [19,20,21]. Decreased sample preparation time and development of more robust sample preparation and analysis methodologies would be beneficial for translational research, lowering the barrier for clinical implementation of biomarker research findings.

## 4. Systemic Multi-Omics Studies of OA-Associated Conditions

Given the time and financial costs, as well as the novelty of developing technologies involved in multi-omics research using human tear samples, prior research has largely focused on systemic studies. These earlier investigations relied on more readily accessible specimens such as serum, plasma and nasal fluid. Serum metabolomics analysis has previously been used to study non-seasonally linked house-dust mite-induced Allergic Rhinitis (AR) [22]. This study found that the arginine and proline metabolism pathway activity was increased in AR vs. healthy controls (HCs), as indicated by increased expression of metabolites such as sarcosine and ornithine [22]. These findings were also shown in a study of serum metabolites in asthmatic children, indicating that arginine and proline metabolic activity is significant in multiple types of atopy [22,23]. This metabolic activity produces intermediates such as nitric oxide and is involved in cellular respiration and inflammation in atopic individuals [22].

Ma et al. corroborated these findings by observing that inflammation was upregulated in perennial AR patients through targeted detection of metabolites indicative of arachidonic acid metabolism in serum samples [24]. Arachidonic acid metabolism generally refers to lipid-mediated inflammatory pathway activation, indicated by dysregulation of lipids. In a study by Xie et al. and another study on non-seasonal AR metabolomics by Zheng et al., arachidonic acid metabolic activity was shown to decrease following immunotherapy, thus indicating that it is a crucial component to metabolism-mediated inflammation in both seasonal and non-seasonally linked AR [22,24,25]. Furthermore, Zhou et al. investigated differences in metabolic activity in AR patient serum occurring during the peak allergy season compared to the off-peak season, finding that increased inflammation during the peak season may be linked to increased energy expenditure through activation of the Krebs cycle [26]. Findings from this study are greatly limited by the lack of an HC group and thus cannot be reliably used to predict seasonal changes occurring solely due to AR. These systemic observations contrast with more localised molecular studies, which focus on protein and miRNA expression changes occurring on the ocular surface.

Proteins involved in symptom expression on the ocular surface of disorders such as keratoconus, DED, AR and the more severe forms of OA have been linked to dysregulated expression of miRNAs [27,28,29,30]. The most commonly used methods for miRNA analysis include next generation sequencing (NGS), real-time quantitative polymerase chain reaction (RT-qPCR) and microarray. Due to the novelty of the field, each method may be applicable depending on the desired outcomes of the research.

Emerging studies on miRNA changes in OA are limited, while studies of AR are more abundant. Prior research has shown that miR-133b downregulation may be a predictive biomarker for allergic inflammation identified in murine models of AR [30]. Serum IgE expression in AR-affected mice was significantly reduced in response to treatment with a miR-133b mimic, which was shown to regulate the expression of TNF-α, IFN-γ, IL-4, IL-5 and IL-10 in response, thereby suggesting that this miRNA has an anti-inflammatory role in murine models of AR when upregulated [30]. Human nasal mucosal samples have been used for microarray analysis to assess differential expression of miRNAs in perennial AR sufferers compared to HC, identifying a total of nine differentially expressed miRNAs [27]. miR-7 and miRPlus-E1194 were increased, while miR-498, miR-187, miR-874, miR-143, miR-886-3p, miR-224 and miR-767-5p were decreased among perennial AR sufferers when compared to HC [27]. These miRNAs are theorised to regulate allergic and inflammatory biopathways; however, due to the novelty of their findings, not all miRNAs have been mapped to gene–protein interactions at this time. Nevertheless, identifying biomarkers of disorders such as AR and OA in the bodily fluids typically associated with the most prominent sites of inflammation provides an important starting point for diagnostic and therapeutic discovery.

**Figure 1:**
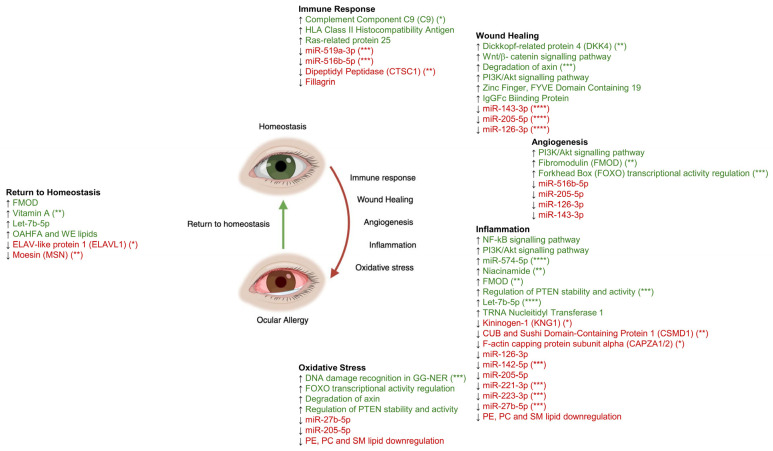
Biopathway from immune response to homeostasis occurring on the ocular surface, including all biomarkers identified in the focal studies and how they relate to the key phases of the allergic response. Biomarkers included in this figure were selected based on in-study significance (*p* < 0.05) and biological relevance in the context of this review. Asterisks were used to denote significance based on *p*-value. * = 0.05–0.02; ** = 0.02–0.01; *** = 0.005–1.0 × 10^−1^; **** ≤1.0 × 10^−1^. Green/upward arrows represent upregulation between OA and HC, while red/downward arrows represent downregulation. PC: Phosphatidylcholine; PE: phosphatidylethanolamine; SM: sphingomyelin.

When investigating the findings from recent -omics publications simultaneously [14,15,31,32,33], a clear pathway from a healthy ocular surface to an itchy, red and inflamed one emerges. Key tear-based biomarkers integrated into this proposed ocular allergy biopathway model are summarised in Table 1. A cycle from allergen exposure to homeostasis is shown in Figure 1. Following allergen exposure, an immune response is stimulated, leading to the release of signalling molecules such as cytokines, as reported in the literature.

## 5. Immune Response

The immune response is a crucial function of the body, defending against pathogen invasion. Hypersensitivity refers to an overreaction of the immune response, resulting in cytotoxicity, inflammation or allergy. In OA, the immune response is mediated by the release of histamine following allergen-triggered mast cell activation. The release of histamine triggers symptoms such as redness, itchiness and swelling of the ocular surface and eyelids [21]. Upon allergen exposure on the ocular surface, a protein and cytokine signalling storm triggers expression of miRNAs and proteins responsible for angiogenesis to encourage formation of new capillaries on the ocular surface [46]. Many allergy biopathway-associated proteins have been identified via proteomics analysis, likely because tears are not collected immediately after an allergy attack is triggered, but rather amid allergy seasons. As a result, few significant differences have been reported in the literature depicting biomarker expression in the early stages of the post-allergic response, as hypothetically they may have been flushed away from the ocular surface to be replaced by cell-signalling and symptom-triggering mediators. HLA class II histocompatibility antigen DRB5 beta chain (HLA DRB5) was shown to be upregulated in a discovery study of OA using human tears collected via Schirmer strip [32]. This finding indicated an increase in antigen presentation to CD4 T cells during early allergy, potentially contributing to the amplification of allergic inflammation [34]. Immune proteins Dipeptidyl peptidase (CTSC1) and Complement component c9 (C9) were also upregulated in OA [15]. CTSC1 and C9 regulate innate immunity, neutrophil activation and inflammation in the tears, leading to an increase in transport of allergy mediators to the site of allergen exposure to clear away irritation-triggering allergy peptides on the ocular surface [35,36]. These allergic peptides are likely the root cause behind OA symptoms such as itching, which in turn may lead to OA sufferers rubbing their eyes to self-regulate and hence inducing tissue-level injury on the ocular surface.

## 6. Wound Healing

Wound healing is theorised to be induced on the ocular surface in response to allergy-associated eye rubbing and irritation. It is the process whereby inflammation and angiogenesis are increased to facilitate transport of cytokine, chemokine and protein mediators to sites of injury or “wounds” on the ocular surface, potentially caused by eye rubbing [52]. Differential expression of cell mediators (such as proteins, lipids, miRNAs) and activation and inhibition of various biopathways, are crucial for ensuring wound healing on the ocular surface. The ultimate goal of wound healing pathways is to enhance progression towards homeostasis on the ocular surface, in response to allergen exposure and inflammation.

Wound healing, inflammation and angiogenesis have been shown to be upregulated on the ocular surface in response to eye rubbing in previous studies of keratoconus via the differential expression of cytokines, chemokines and proteins [53]. Tears are not typically collected directly after eye rubbing, yet may still show evidence of wound healing and cell structure repair on the ocular surface through metabolic remnants from biopathways such as the Wnt/β-catenin signalling pathway—crucial for epithelial cell proliferation and tissue regeneration; phosphoinositide 3-kinase (PI3K)/the serine/threonine protein kinase B (Akt) signalling pathway (PI3K/Akt pathway)—important for cell survival, inflammation and angiogenesis; and the nuclear factor kappa-light-chain-enhancer of activated B cells (NF-κB) biopathway, which regulates inflammatory and immune responses [15]. Collectively, these pathways highlight the connections between the biological mechanisms that are most impacted by sustained inflammation resulting from eye rubbing-induced mechanical stress on the ocular surface in conditions such as OA and keratoconus.

The Wnt/β-catenin and PI3K/Akt signalling pathways are hypothesised to work in conjunction, as a variety of overlapping factors suggest an interlinked response to injury [54] on the ocular surface. In the skin, the Wnt/β-catenin pathway initiates the proliferative phase of wound healing, in which new cells are generated to replace injured cells [54]. This is further suggested by increased expression of Zinc Finger, FYVE Domain Containing 19 (ZFYVE19), a protein linked to cytokinesis and cell division regulation [32]. Cell transport occurs through formation of new blood vessels, also known as angiogenesis. The PI3K/Akt signalling pathway triggers both inflammation and angiogenesis, leading to further activation of the proinflammatory NF-κB biopathway [45].

The proliferation-stimulating Wnt/β-catenin pathway was highlighted through differential expression of a number of cell mediators, including Dickkopf-related protein 4 (DKK4) in OA [15]. DKK4 was found to inhibit Wnt expression and signalling via functional analysis, thereby causing decreased expression of lipids on the ocular surface [37]. Dysregulated lipid expression could have severe impacts on the tear film, affecting factors such as tear film stability, surface tension, dry eye and poor visual health outcomes. Lipid expression dysregulation may then lead to increased inflammation on the ocular surface [37], which can be detected by metabolomic and proteomics discovery analyses to identify inflammatory biopathway activation. Vera-Montecinos et al. identified increased expression of the mucin-like protein IgGFc-binding protein (FCGBP), hypothesising that the involvement of the protein in mucosal epithelial defence may thus fulfil a cytoprotective and anti-inflammatory role on the ocular surface [32]. Furthermore, enrichment analysis of significant metabolites identified by Aydin et al. posited that decreased degradation of axin protein across both the peak allergy season and the off-peak season in OA sufferers could be due to the role of axin as a key component that inhibits the Wnt pathway [14]. Therefore, a net increase in Wnt pathway signalling, and thus wound healing, is potentially detectable in the tears of OA sufferers.

The Wnt/β-catenin pathway may also be inhibited by expression of miRNAs, as shown in miR-143-3p validation-focused mimic models in corneal epithelial cells [44]. However, mir-143-3p was shown to be downregulated in OA and would not have this upregulating effect on wound healing mediator expression [31]. Additionally, differentially expressed miR-205 and miR-126 were shown to have net upregulating roles in wound healing [31]. Overall, differential expression of factors involved in upregulated wound healing on the ocular surface were also shown to be involved in inflammation and angiogenesis.

## 7. Angiogenesis

In OA, angiogenesis may occur as a result of an increased need for oxygen and nutrient supply on the ocular surface in response to mechanical stress and injury. Newly formed capillaries resulting from increased angiogenesis are used as transport channels from the blood to the ocular surface to disseminate more immune and inflammatory proteins and miRNAs, while appearing on the ocular surface as redness and irritation [46]. Dysregulated expression of Vascular Endothelial Growth Factor (VEGF) proteins has been linked to differential expression of miR-516b-5p, miR-205-5p and miR-126-3p in OA via functional analysis [31]. VEGF proteins stimulate angiogenesis by forming complexes of multiple specialised VEGF proteins (such as VEGF-A, VEGF-B and VEGF-E) into VEGF receptors (VEGFR), which are capable of acting as a major transducer for several angiogenesis-triggering biopathways, including PI3K/Akt signalling pathways [55,56]. It is theorised that miR-516b-5p, miR-205-5p and miR-126-3p may regulate expression of VEGF proteins and thus are able to effect activation of angiogenesis on the ocular surface [46,47,48]. It is proposed that other differentially expressed miRNAs may also be involved in angiogenesis [31]; however, further research needs to be undertaken to investigate this hypothesis.

Proteomics findings identified upregulation of Fibromodulin (FMOD)—crucial for organising extracellular matrix and collagen—in OA sufferers’ tears, likely linked to increased angiogenesis and wound healing [15]. Metabolic analysis also identified upregulation of Forkhead Box (FOXO) proteins occurring in OA vs. HC, irrespective of season [14], which has been linked to angiogenesis and oxidative stress in the literature [57]. Given that the regulation of FOXO was increased in OA sufferers perennially [14], it is likely involved in cross-seasonal upregulation of angiogenesis, while also playing a key role in regulation of oxidative stress due to inflammation and pollen exposure on the ocular surface [15,46,50]. Angiogenesis has therefore been identified as a crucial biopathway on the ocular surface of OA sufferers alongside inflammation, as miRNAs work to regulate proinflammatory protein expression in response to allergen invasion.

## 8. Inflammation

Inflammation is a key driver of OA, occurring in response to allergen exposure. Through mast cell activation and the subsequent release of inflammatory mediators, inflammation facilitates vasodilation, tissue oedema (swelling) and attraction of immune cells to the site of allergen exposure via chemotaxis [21]. Inflammation thereby underpins the primary physiological signs of redness and swelling in OA [15]. Differentially expressed proteins provide the greatest evidence of inflammation on the ocular surface through dysregulation of Kininogen-1 (KNG1) and CUB And Sushi Multiple Domains 1 (CSMD1) [15], TRNA Nucleotidyl Transferase 1 (TRNT1), Ras-related protein 25 (RAB25) and FCGBP [32], culminating in a net increase in inflammation on the ocular surface. Additionally, f-actin capping protein subunit alpha (CAPZA 1/2) was downregulated perennially in OA [15]. This was likely due to cell breakdown caused by inflammation and allergy-associated eye rubbing [38]. Other downregulated proteins (likely mediated by miRNAs), such as KNG1 and CSMD1 [15], were shown to have roles in inflammation, as well as wound healing and angiogenesis [39,40,41,42].

The literature highlights a potentially significant role of miRNAs in inflammation through upregulation of miRNAs from the let-7 family, as well as downregulation of miR-126-3p, miR-142-5p, miR-205-5p, miR-221-3p and miR-223-3p [31]. Downregulated miRNAs in OA were linked to roles in the net upregulation of inflammation via modified inflammatory cytokine expression [31]. Inflammatory cytokine expression was shown to be upregulated in OA tears by Gijs et al., namely, by the expression of proteins Chemokine (C-C motif) ligand 25 (CCL25), C-C motif chemokine 7 (CCL7), Contactin-associated protein-like 2 (CNTNAP2), Interferon gamma (IFNG), Interleukin-17F (IL17F), Interleukin-5 receptor subunit alpha (IL5RA) and tumour necrosis factor receptor superfamily member 13C (TNFRSF13C) [33]. The NF-κB pathway also enacts its proinflammatory function by modifying the expression of cytokines [58]. This pathway has been activated and inhibited by various differentially expressed mediators, including miR-574-5p, niacinamide and theanine [14,31]. Other inflammatory biopathways have been identified as being activated on the ocular surface of OA sufferers, including the PI3K/Akt pathway [31,32].

The PI3K/Akt pathway influences inflammation on the ocular surface through fibroblast activation [45]. miR-27b-5p was downregulated among OA sufferers [31] and may lead to increased inflammation on the ocular surface by promoting fibroblast activation, as shown in the literature [45]. Upregulated FMOD expression corroborated this finding [45]. The proinflammatory effect occurs via activated fibroblasts, which trigger a cytokine cascade on the ocular surface [45]. Taken together, findings in this article suggest that the PI3K/Akt pathway plays a variety of roles on the ocular surface of OA sufferers, including inflammation, angiogenesis and wound healing [45]. However, sustained inflammation for long periods of time (e.g., the duration of spring–summer) may lead to increases in oxidative stress on the ocular surfaces of OA sufferers.

## 9. Oxidative Stress

Oxidative stress occurs most commonly as a result of increased Reactive Oxygen Species (ROS) and inflammation, as shown in studies of asthma and OA [59]. On the ocular surface, oxidative stress may lead to increased apoptosis, DNA damage and immune system hyperactivity [50]. Oxidative stress biopathways are mediated by expression of Forkhead Box (FOXO) in injured and inflamed cells, as shown in validation-focused literature [57]. In a study by Wan et al., a hypoxic environment with periodic reoxygenation was used while culturing human umbilical vein endothelial cells (HUVECs) to stimulate oxidative stress conditions [57]. The expression levels of FOXO were found to be positively associated with ROS and expression of angiogenesis mediators such as VEGF proteins [57]. FOXO transcription was also found to be triggered by the PI3K/Akt pathway [57]. Ultimately, FOXO was found to regulate oxidative stress in vitro by increasing secretion of antioxidants [57]. As a result, decreased oxidative stress via FOXO transcriptional regulation helps return inflamed cells to homeostasis following stress-inducing events such as allergic reactions.

At its peak, inflammation and angiogenesis spur on oxidative stress biopathways on the ocular surface, while downregulating choline-derived lipid species. Downregulation of crucial lipids can affect tear film stability, increasing evaporation off the ocular surface [51]. The ocular surface becomes irritated, red and itchy, which in turn leads to patient discomfort. Likely in response, an increase in expression of FCGBP has been detected in allergy sufferers’ tears’ [32], leading to a net anti-inflammatory effect [60]. To supplement this, many OA sufferers additionally resort to the use of eye drops, over-the-counter medications or, most concerningly, eye rubbing, to manage these symptoms [61].

In an effort to degrade and clear away allergic peptides and other irritants, metabolic pathways of amino acid degradation, oxidative stress and anti-inflammation may be increased on the ocular surface. The implication of these findings is that while anti-inflammatory and homeostatic mediators become more evident in proteins and lipids during off-peak season, there are still remnants of inflammation and oxidative stress in the tears. Given the increase in oxidative stress perennially, it is hypothesised that this oxidative stress-triggered angiogenesis does not boost inflammatory pathways as previously shown, but instead induces a return to homeostasis.

## 10. Return to Homeostasis

The final step of the OA cycle, which consists of immune response, wound healing, angiogenesis, inflammation and oxidative stress, is a return to homeostasis (Figure 1). Protein, miRNA, metabolite and lipid markers indicating this return to homeostasis were identified by this review.

Differentially expressed homeostasis proteins in OA vs HC included FMOD, Moesin (MSN) and ELAV-like protein 1 (ELVAL1) [15]. FMOD upregulation and its role in angiogenesis were previously discussed; however, this protein has also been linked to a return to homeostasis through its modification of TGF-β activity, thus giving it an anti-inflammatory effect [62]. MSN and ELAVL1 were both downregulated, indicating that homeostasis pathways were downregulated among OA sufferers as they were in the midst of a high-allergen exposure period [15]. Let-7b-5p was also shown to be involved in return to homeostasis following an inflammation flare-up [43]. This miRNA was theorised to play an anti-inflammatory role on the ocular surface to facilitate a return to homeostasis [31]. This is supported by metabolomics findings, which highlight significant upregulation of vitamin A in OA [14]. Vitamin A has been linked to tear film maintenance and anti-inflammatory roles [49], which are likely to compensate for downregulation of tear film-stabilising lipids in OA tears [31].

The current landscape of the literature has laid the foundation for a complex understanding of the various metabolic biopathways being activated and inhibited on the ocular surface in OA, as well as the protein–protein interactions, miRNA modifications and lipidomic changes occurring in the tear film.

## 11. Connections to Comorbidities

A study by Gijis et al. from 2023 comparing OA, keratoconus and OA + keratoconus groups’ tear proteomes found that there was some overlap in the conditions that indicated a shared underlying pathophysiology [33]. Namely, expression of LGALS9 (galectin-9) was upregulated in both keratoconus and the OA + keratoconus groups, indicating an involvement with immunomodulatory effects and corneal disease [63,64]. In the OA-specific group, upregulation of proteins such as TNFRDF4 (OX40) and CCL13 present as strong biomarkers of T-cell-driven allergic inflammation and chronic inflammation and allergic disease, respectively [33]. Furthermore, downregulation of ectodysplasin A receptor (EDAR) was demonstrated in the OA group [33]. Typically, EDAR is abundantly expressed in the ocular surface and surrounding meibomian and lacrimal glands. Downregulation has been observed in other ocular disorders, such as DED, and has been linked to various forms of atopy [65].

Bridging the gap between OA and DED, a 2024 study by Jiao et al. explored targeted expression of Tear Lymphotoxin-α (TLα), Immunoglobulin E (IgE) and Matrix Metalloproteinase-9 (MMP9) in Seasonal/Perennial Allergic Conjunctivitis-Associated Dry Eye (S/PAC-DE) and DED [66]. TLα, IgE and MMP9 were all significantly elevated in S/PAC-DE compared to DED, indicating a likely involvement of the three proteins in OA pathophysiology that is distinct from, or compounded by, co-morbid DED. This possible association is supported by de la Fuente et al. in 2022, who found that MMP9 expression is predictive for ocular surface inflammation among a cohort of ocular disorders, including cataracts, glaucoma, OA, DED and meibomian gland dysfunction [67]. These findings underscore the value of highly novel emergent biomarker research, setting the tone for broad-scope multi-omics investigations into OA pathophysiology that are yet to come.

## 12. Limitations

A key limitation of the unified biopathway model of OA presented in this article is the lack of cross-comparability of existing studies. This drawback arises from substantial methodological variations between studies, including sample size, tear sample collection method (Schirmer strip vs. glass microcapillary flow) and analytical methods (mass spectrometry, next generation sequencing, etc.). Differences in tear collection methods alone may introduce confounding factors such as reflex tearing, cellular contamination and analyte retention, likely influencing downstream protein, lipid, metabolite and miRNA profiles. A detailed comparison of tear collection methods and analytical techniques has previously been presented in a dedicated methods-focused review [21]. Furthermore, analytical approaches range from multiplex immunoassays and automated electrophoretic technologies to mass-spectrometry-based workflows, each with distinct sensitivity, dynamic ranges and sample preparation requirements [21].

Consequently, there is little consistency in reported differentially expressed analytes across studies, restricting the robustness of cross-study comparisons. This inconsistency highlights a critical gap in the literature that would best be addressed through a pivotal large-scale, longitudinal study of OA pathophysiology using standardised tear collection and analytical methodologies. The high analyte recovery rates of glass microcapillary flow and the sensitive detection rates of mass spectrometry lend themselves to being a gold standard method for conducting discovery assays to investigate biomarkers of ocular allergy.

While the novelty of human tear multi-omics research has historically impacted the feasibility of such studies, recent technological advances now enable high-throughput, time- and cost-efficient multi-omics workflows, lowering previous financial and logistical barriers to comprehensive OA research.

## 13. Conclusions

In this review, we sought to link potential biomarkers of OA in the proteome, lipidome, metabolome and miRNA landscape of the ocular surface using human tears for the first time to develop a model of OA biopathway activation, broken down into steps from immune response to return to homeostasis. Fibromodulin (FMOD), Forkhead Box (FOXO), and miR-205-5p emerged as key biomarkers representing the interconnected pathways of inflammation, wound healing and angiogenesis in OA. Their differential expression highlights the potential of human tear multi-omics research to objectively characterise disease severity and identify novel targets for improved diagnosis and therapy. Additionally, we aimed to review emerging technical research on extraction and analysis methodologies, as well as adjacent findings in other ocular disorder studies, including DED and keratoconus. Ample room remains for additional studies to validate findings and assess their diagnostic and therapeutic capacities to fulfil the overarching aim of identifying biomarkers of OA to improve health outcomes for allergy sufferers.

## Figures and Tables

**Table 1:** This table summarises proteins, microRNAs (miRNAs), metabolites and lipid classes reported to be differentially expressed in the tears of individuals with ocular allergies across the studies reviewed. Biomarkers were included based on reported statistical significance within the original publications (*p* < 0.05) and biological plausibility within the proposed ocular allergy biopathway model. Where directionality of expression was not consistently reported across studies, biomarkers are described as dysregulated.

Analyte	Biomarker	Regulation in OA	Primary Biopathway(s)	Proposed Role on the Ocular Surface
Proteins	HLA class II histocompatibility antigen DRB5 beta chain (HLA-DRB5)	Upregulated	Immune response	Enhanced antigen presentation to CD4^+^ T cells, contributing to amplification of allergic inflammation [32,34]
	Dipeptidyl peptidase (CTSC1)	Upregulated	Immune response, inflammation	Regulation of innate immunity and neutrophil activation, facilitating clearance of allergenic peptides [15,35,36]
	Complement component 9 (C9)	Upregulated	Immune response, inflammation	Activation of complement-mediated inflammatory responses [31,35,36]
	Fibromodulin (FMOD)	Upregulated	Wound healing, angiogenesis, inflammation	Extracellular matrix organisation, fibroblast activation, angiogenesis and modulation of inflammatory responses [15]
	IgGFc-binding protein (FCGBP)	Upregulated	Inflammation, homeostasis	Mucosal epithelial defence with cytoprotective and anti-inflammatory functions [32]
	Zinc Finger, FYVE Domain Containing 19 (ZFYVE19)	Upregulated	Wound healing	Regulation of cytokinesis and epithelial cell proliferation [32]
	Dickkopf-related protein 4 (DKK4)	Upregulated	Wound healing	Inhibition of Wnt signalling [15,37]
	F-actin capping protein subunit alpha (CAPZA 1/2)	Downregulated	Cytoskeletal integrity, inflammation	Indicative of cell structure disruption due to inflammation and mechanical stress (eye rubbing) [15,38]
	Moesin (MSN)	Downregulated	Homeostasis	Cytoskeletal organisation and maintenance of epithelial stability [15]
	ELAV-like protein 1 (ELAV1)	Downregulated	Homeostasis	Post-transcriptional regulation of inflammatory mediators [15]
	TRNA Nucleotidyl Transferase 1 (TRNT1)	Dysregulated	Inflammation	Regulation of tRNA maturation and cellular stress responses [32]
	Ras-related protein 25 (RAB25)	Dysregulated	Inflammation	Regulation of vesicle trafficking and epithelial polarity [32]
	Kininogen-1 (KNG1)	Dysregulated (downregulated in OA)	Inflammation, wound healing	Regulation of inflammatory cascades and vascular permeability [15,39,40,41,42]
	CUB And Sushi Multiple Domains 1 (CSMD1)	Dysregulated (downregulated in OA)	Inflammation, angiogenesis	Modulation of complement activation and inflammatory signalling [15,39,40,41,42]
miRNAs	let-7 family (e.g., let-7b-5p)	Upregulated	Inflammation, homeostasis	Anti-inflammatory regulation and facilitation of return to homeostasis [31,43]
	miR-143-3p	Downregulated	Wound healing	Reduced inhibition of Wnt/β-catenin signalling, favouring epithelial proliferation [31,44]
	miR-27b-5p	Downregulated	Inflammation	Promotion of fibroblast activation and proinflammatory cytokine release [31,45]
	miR-142-5p	Downregulated	Inflammation	Modulation of immune cell activation and cytokine expression [31]
	miR-221-3p	Downregulated	Inflammation	Regulation of inflammatory signalling pathways [31]
	miR-223-3p	Downregulated	Inflammation	Control of innate immune responses [31]
	miR-205-5p	Dysregulated	Wound healing, angiogenesis, inflammation	Regulation of epithelial repair, VEGF signalling and inflammatory mediator expression [31,46,47,48]
	miR-126-3p	Dysregulated	Angiogenesis, inflammation	Modulation of VEGF-driven angiogenesis and endothelial function [31,46,47,48]
	miR-516b-5p	Dysregulated	Angiogenesis	Regulation of VEGF expression and neovascularization [31,46,47,48]
	miR-574-5p	Dysregulated	Inflammation	Modulation of inflammatory signalling pathways, including regulation of NF-κB-associated cytokine expression [31]
Metabolites/Pathways	Vitamin A	Upregulated	Homeostasis, anti-inflammation	Tear film maintenance and anti-inflammatory compensation [14,49]
	FOXO regulation	Upregulated	Oxidative stress, angiogenesis	Regulation of oxidative stress responses and antioxidant secretion [14,46,50]
	Niacinamide/Vitamin B3	Dysregulated	Inflammation, oxidative stress	Anti-inflammatory and antioxidant activity [14]
	Theanine	Dysregulated	Inflammation, oxidative stress	Anti-inflammatory and antioxidant activity [14]
Lipids	Choline-derived lipids	Downregulated	Tear film stability, oxidative stress	Reduced tear film stability and increased evaporation [14,51]
	Phosphatidylcholine/Phosphatidylethanolamine	Downregulated	Tear film integrity	Altered lipid layer composition contributing to inflammation and dry eye [14,51]

## Data Availability

No new data were created or analysed in this study. Data sharing is not applicable to this article.
